# Justiça reprodutiva e desigualdades interseccionais: uma leitura crítica da coletânea organizada por Brandão, Lowenkron & Carneiro

**DOI:** 10.1590/0102-311XPT242825

**Published:** 2026-03-09

**Authors:** Stephania Klujsza

**Affiliations:** 1 Universidade Federal Fluminense, Niterói, Brasil.

A obra organizada por Elaine Brandão, Laura Lowenkron e Rosamaria Carneiro marca a necessidade de ampliarmos o olhar para os direitos reprodutivos de meninas, mulheres e pessoas que gestam ^1^. Prefaciado por Lúcia Xavier e Mônica Sacramento, da organização não-governamental (ONG) Criola - entidade de extrema relevância e de intensa ação política para os debates sobre os direitos das mulheres negras no Brasil -, o livro apresenta diversos contextos de pesquisas de campo acerca do exercício das maternidades, evidenciando como os marcadores sociais da diferença produzem desigualdades.

Ao reunir pesquisas que atravessam diferentes contextos, a coletânea destaca que pensar direitos reprodutivos exige compreender como gênero, raça, classe, idade, território e corporalidades não são marcadores isolados, mas elementos que se articulam interseccionalmente na produção de vulnerabilidades e privilégios ^2^. Assim, as pesquisas ali reunidas demonstram que as experiências reprodutivas não podem ser analisadas de forma homogênea: elas são moldadas por sistemas de opressão que operam simultaneamente e estruturam o acesso - ou a negação - de direitos.

Os capítulos dialogam diretamente com o campo da justiça reprodutiva, abordagem que ultrapassa a noção liberal de “escolha” para situar a reprodução dentro de condições materiais, políticas e sociais concretas. Justiça reprodutiva significa não apenas garantir o direito de gestar ou não gestar, mas assegurar que todas as pessoas possam fazê-lo com dignidade, segurança e autonomia, livres das violências institucionais que historicamente atingem, sobretudo, mulheres negras, indígenas, pobres e periféricas. O livro revela como a reprodução é atravessada por relações de poder que estruturam o Estado, as políticas públicas e as práticas cotidianas dos serviços de saúde. Segundo Brandão & Cabral ^3^ (p. 7):

“*Não se pode falar em autonomia (autodeterminação) reprodutiva quando há obstáculos estruturais que impedem tais escolhas, ou ainda, que perpetuam a desigualdade social, racial e de gênero. Tal compreensão implica o descentramento da responsabilidade das próprias mulheres por sua (in)capacidade de gerir sua vida sexual e reprodutiva; e obriga o Estado a ampará-las em algo que alude à reprodução da vida em sociedade*”.

A seguir, apresento uma breve reflexão sobre cada capítulo, com o intuito de ajudar o leitor que pretende investigar seus interesses mais específicos.

Em *Políticas dos Corpos, (in)Justiça Reprodutiva e o Sistema Moderno Colonial de Gênero*, capítulo que abre o livro, Emanuelle Goés discute como as escolhas reprodutivas e a autonomia corporal das mulheres estão profundamente atravessadas por processos históricos, políticos e coloniais, marcados por opressões de raça, gênero e classe. Goés apresenta exemplos históricos e contemporâneos que evidenciam as formas de injustiça reprodutiva. Segundo a pesquisadora, o discurso dos “direitos reprodutivos universais” continua a reforçar desigualdades raciais e de gênero, tratando determinados corpos como descartáveis em nossa sociedade. Nesse contexto, a justiça reprodutiva surge como uma estratégia política que amplia a noção de direitos reprodutivos ao articular corpo, comunidade e mundo.

No segundo capítulo, Camila Fernandes analisa a maternidade como um campo de disputas teóricas, políticas e sociais atravessado por gênero, classe e raça. Em *Mães em Disputa: Diálogos entre os Estudos sobre Maternidades, Justiça Reprodutiva e o Campo dos Cuidados*, a autora apresenta as discussões das desigualdades reprodutivas e das políticas do cuidado e analisa como o feminismo negro denuncia os limites do feminismo branco que ignorou as especificidades das experiências das maternidades negras. Evidencia-se, assim, que a maternidade nunca deixou de ser um terreno de disputas onde se confrontam opressão e resistência, revelando a necessidade de uma perspectiva interseccional para pensar justiça reprodutiva.

Fabiana Santos Lucena e Carmen Simone Grilo Diniz apresentam, no terceiro capítulo, a problemática da morte materna, e buscam compreender como se dá a atuação, as possibilidades e limitações dos Comitês de Mortalidade Materna (CMMs) nos estados do Sudeste brasileiro. Em *Justiça Reprodutiva e Morte Materna: Ascensão e Queda da Atuação dos Comitês de Mortalidade Materna*, as autoras apresentam um estudo documental, com dados de 2018 a 2022, da Região Sudeste, e analisam como os comitês se organizam. É importante ressaltar que, entre os dados apresentados, as mulheres negras têm escores expressivamente superiores aos das brancas, e isso diz do racismo na assistência a que está submetida a população negra. Finalizam o capítulo questionando se os CMMs ainda são um instrumento importante para a redução da morte materna no país ou se deve-se pensar em novas formas de enfrentamento a essas mortes, na sua maioria evitáveis.

A mortalidade também é abordada no capítulo 4, onde Sílvia Guimarães e Catherine Alès trazem a discussão fundamental das violências vivenciadas pela população Sanöma/Yanomami em *Mães Sanöma/Yanomami: A Tragédia da Mortalidade Infantil e a (In)justiça Reprodutiva*. As autoras revelam que os sanöma enfrentam altos índices de mortalidade infantil de crianças com menos de um ano por situações que seriam evitadas com assistência à saúde adequada. Nesse contexto, Guimarães & Alès discutem a forma como a experiência de mulheres indígenas apresenta particularidades, criando problemas e vulnerabilidades próprias. O racismo se apresenta como determinante para a falta de acesso às tecnologias de cuidados, tirando a possibilidade dessas mulheres garantirem a vida delas mesmas e de seus filhos.

“O problema é cultural” é, talvez, a frase mais impactante do capítulo 5, onde Jaciane Milanezi analisa como mulheres haitianas migrantes, usuárias de uma unidade básica de saúde na cidade de São Paulo, tiveram suas experiências reprodutivas marcadas por estigmas e avaliações institucionais. A pesquisadora apresenta sua etnografia sobre fluxos migratórios e reprodução de mulheres haitianas, deixando evidente as hierarquias reprodutivas relacionadas à raça, classe e nacionalidade e como as burocracias do Estado operam dimensões morais na gestão de populações vulneráveis, reproduzindo desigualdades interseccionais ao condicionar o acesso aos serviços públicos a estigmas, regras institucionais e avaliações comportamentais.

O sexto capítulo do livro é de autoria de Anne Alencar Monteiro. Em *Experiências Transmasculinas e os Desafios Frente à Justiça Reprodutiva*, a autora apresenta sua pesquisa de campo com homens trans e pessoas transmasculinas em suas experiências de gestação. A partir das provocações de Monteiro e das falas de seus entrevistados, fica gritante a realidade de discriminações e barreiras de acesso à saúde que pessoas trans vivenciam em nossa sociedade, o que, muitas vezes, as colocam em situações de extrema vulnerabilidade e as afastam dos serviços de saúde.

Waleska Aureliano traz as questões em torno da maternidade “atípica” no capítulo 7, experiência que a pesquisadora compartilha como mãe de uma criança com deficiência. Em *(Sobre)Viver nas Engrenagens do ‘Sistema’: Maternidade, Deficiência e a Politização dos Afetos*, Aureliano nos apresenta como o conceito de justiça reprodutiva se relaciona com a experiência e as dificuldades enfrentadas por mulheres com filhos com deficiência. A partir de uma etnografia em ambientes digitais, a autora apresenta as lutas dessas mulheres nos desafios do cuidado, da sociedade, do sistema de saúde e, muitas vezes, o abandono paterno, familiar e comunitário.

Em *Entre Mulheres que Destituem e que São Destituídas: Reflexões sobre a Atuação do Poder Judiciário e a Justiça Reprodutiva em Casos de Destituição do Poder Familiar*, Janaína Gomes apresenta sua pesquisa com profissionais responsáveis por produzir laudos que informam juízes sobre processos de destituição do poder familiar de crianças, usando como pano de fundo um processo em que a mãe perdeu a guarda de sua terceira filha. No oitavo capítulo, a pesquisadora tensiona a ideia de injustiça reprodutiva problematizando situações em que mulheres são julgadas a partir de uma certa moralidade que as compreende como inaptas ao cuidado parental, mesmo quando não há garantia de direitos básicos para essas mulheres e suas famílias pelo Estado.

O livro se encerra com a apresentação do trabalho de pesquisa de Marjorie Murray e Constanza Tizzoni sobre violência em um município na periferia sul de Santiago do Chile. As autoras apresentam como a vida das mulheres e suas famílias é atravessada pelas injustiças reprodutivas em territórios marginalizados. Partindo de uma situação dramática que afeta a vida de uma interlocutora, Murray & Tizzoni, além de questionar a ausência do Estado, fazem uma reflexão importante sobre como a lógica neoliberal afeta a vida das pessoas e acaba por nos impor um “individualismo agêntico” para tentar buscar uma vida melhor.

A obra é fruto de um esforço corajoso e absolutamente necessário das organizadoras para pensarmos a ideia da justiça reprodutiva e das imensas e recorrentes injustiças reprodutivas que abalam os direitos das mulheres e moldam os seus exercícios de maternidade, sempre deixando clara como a interseccionalidade orienta brutalmente as experiências vivenciadas.
